# Supplementary material for: Optimization of Agrobacterium-mediated transformation of commercial heirloom tomato cultivars to develop novel traits via CRISPR/Cas9 genome editing
Source: Planta. 2026 May 21;264(1):3. doi: 10.1007/s00425-026-05024-9 (PMC13194190; doi:10.1007/s00425-026-05024-9)

**Supplemental Data**

##

## Title:

Optimization of *Agrobacterium*-mediated transformation of commercial heirloom tomato cultivars to develop novel traits via CRISPR/Cas9 Genome Editing.

## Authors:

Jordan Oxendine^1,2^, Elsa Ibarra-Reyes^1,2^, Junchi Ma^2^, Carrie Li^2^, Sarah Baron^2^, Allison E. Hwang^2^, Ruiting Wang^2^ and Daniel Rodriguez-Leal^2,^*

## Author information:

^1^Equal contribution

^2^Tomato Lab. Department of Plant Science and Landscape Architecture. University of Maryland, College Park, MD, 20742, US.

## Correspondence:

*[danielrl@umd.edu](mailto:danielrl@umd.edu), phone: +1 (301) 405-0920

**ORCiD:** https://orcid.org/0000-0003-1871-7432

| Table S1. guide RNA sequences used in this study | |
| --- | --- |
| **gRNA id** | **gRNA sequence** |
| *SPG5-g1* | AGAGATCCTTTAATAGTTTC |
| *SPG5-g2* | AACAATAGGGTGGTCTATAA |
| *Br_g1* | GTGGAGAACTCCGATTGCCA |
| *Br_g2* | GATGTGATGACTTCATTACT |

| Table S2. Primers used in this study | | |
| --- | --- | --- |
| **primer id** | **sequence (5´to 3´)** | **purpose** |
| TL0231 | CATTGCGATTCTTGACGACGA | Br genotyping and sequencing |
| TL0143 | GGGAGACTACCCCACTATCCA | Br genotyping and sequencing |
| TL0144 | TTCACGACTTGTCAACCATTG | SP5G genotyping and sequencing |
| TL0199 | GGTTGCTAGGGTTTGGAGCA | SP5G genotyping and sequencing |
| TL0246 | tgtggtctcaATTGAGAGATCCTTTAATAGTTTCgttttagagctagaaatagcaag | SP5G-1 gRNA cloning |
| TL0247 | tgtggtctcaATTGAACAATAGGGTGGTCTATAAgttttagagctagaaatagcaag | SP5G-2 gRNA cloning |
| TL0248 | tgtggtctcaATTGGTGGAGAACTCCGATTGCCAgttttagagctagaaatagcaag | Br-1 gRNA cloning |
| TL0249 | tgtggtctcaATTGGATGTGATGACTTCATTACTgttttagagctagaaatagcaag | Br-2 gRNA cloning |
| TL0250 | tgtggtctcaAGCGTAATGCCAACTTTGTAC | gRNA cloning (universal primer) |

| Table S3. Information on heirloom cultivars used in this study | | | | | |
| --- | --- | --- | --- | --- | --- |
| **Variety** | **Days to Maturity** | **Fruit Color** | **Fruit Size / Shape** | **Growth Habit** | **Notable Characteristics** |
| M82 | 75–80 | Red | Medium, round | Determinate | Standard research cultivar with strong uniformity. |
| Amana Orange | 85–90 | Deep orange | Very large, beefsteak | Indeterminate | Mild, sweet, low-acid; large fruit set. |
| Brandywine Pink | 85–100 | Pink-red | Very large, beefsteak | Indeterminate | Classic Brandywine flavor; potato-leaf trait. |
| Jubilee | 70–80 | Red-orange | Medium, elongated plum | Indeterminate | Sweet, low-acid; consistent coloration. |
| Mortgage Lifter | 80–85 | Pinkish-red | Large, beefsteak | Indeterminate | High-yielding; rich balanced flavor. |
| Red Potato Leaf Brandywine | 80–95 | Red | Large, beefsteak | Indeterminate | Red-fruited potato-leaf Brandywine type. |
| Sunray | 75–80 | Orange | Medium, round | Indeterminate | Smooth, uniform fruits. |

| Table S4. Raw data on tissue culture and transformation experiments | | | | | | | | |
| --- | --- | --- | --- | --- | --- | --- | --- | --- |
| **Vector** | **Cultivar** | **Total # seeds** | **Total # explants** | **Total Infected^a^** | **Total # of shoots** | **Regeneration %^b^** | **Total # rooted** | **Total in soil** |
| pTL0047 | M82 | 150 | 86 | 86 | 29 | 33.70% | 35 | 0 |
| pTL0047 | Amana Orange | 150 | 33 | 33 | 11 | 33.30% | 1 | 0 |
| pTL0047 | BW. Pink | 150 | 34 | 34 | 3 | 8.80% | 1 | 0 |
| pTL0047 | Jubilee | 150 | 29 | 29 | 1 | 3.40% | 1 | 0 |
| pTL0047 | Mortgage Lifter | 150 | 38 | 38 | 10 | 26.30% | 2 | 0 |
| pTL0047 | RP-BW | 150 | 80 | 80 | 33 | 41.20% | 8 | 0 |
| pTL0047 | Sunray | 150 | 33 | 33 | 8 | 24.20% | 0 | 0 |
| pTL0151 | M82 | 300 | 259 | 64 | 49 | 76.60% | 22 | 14 |
| pTL0151 | Jubilee | 250 | 276 | 132 | 30 | 22.70% | 11 | 0 |
| pTL0151 | Sunray | 250 | 136 | 100 | 30 | 30.00% | 10 | 6 |
| pTL0153 | M82 | 250 | 150 | 67 | 23 | 34.30% | 11 | 5 |
| pTL0153 | Jubilee | 250 | 200 | 69 | 23 | 33.30% | 6 | 2 |
| pTL0153 | Sunray | 250 | 125 | 101 | 35 | 34.70% | 2 | 1 |
| pTL0153 | Amana Orange | 300 | 99 | 80 | 17 | 21.30% | 30 | 6 |
| pTL0153 | BW. Pink | 250 | 97 | 52 | 26 | 50.00% | 9 | 0 |
| ^a^Number of explants that were suitable for infection (e.g. not damaged/contaminated).  ^b^ Percentage of # shoots observed divided by total infected explants. | | | | | | | | |

| Table S5. Two proportion Z-test test on transformation efficiency between the 3 vectors used in this study | | | | | |
| --- | --- | --- | --- | --- | --- |
| **Genotype** | **pTL0047** | **pTL0151** | **pTL0153** | **Two proportion Z-test**  **P-value (pTL0047 vs pTL0153)** | **Two proportion Z-test**  **P-value (pTL0151 vs pTL0153)** |
| M82 | 29/86 | 49/64 | 23/67 | 1 (ns) | 0.000001*** |
| Jubilee | 1/29 | 30/132 | 23/69 | 0.0039*** | 0.105168 (ns) |
| Sunray | 8/33 | 30/100 | 35/101 | 0.3694 (ns) | 0.480689 (ns) |
| ^a^Number of shoots divided by total functional explants. | | | | | |

| Table S6. Comparing timeline for transformation between pTL0151 and pTL153 | | | | | | |
| --- | --- | --- | --- | --- | --- | --- |
| **Genotype** | **Days to rooting media (pTL151)** | **Days to rooting media (pTL0153)** | **Difference (days)** | **Days to transplanting (pTL0151)** | **Days to transplanting (pTL0153)** | **Difference (days)** |
| M82 | 62 | 64 | 2 | 19 | 11 | 8 |
| Jubilee | 78 | 53 | 25 | NA^a^ | 26 | NA |
| Sunray | 61 | 56 | 5 | 15 | 10 | 5 |
| ^a^Rooted plants were lost due to contamination in glass jars and did not recover properly for transplanting. | | | | | | |

| Table S7. Comparison of rooting efficiencies on different growth regulators in Mortgage lifter heirloom | |
| --- | --- |
| **Root media treatments ^a^** | **Mortgage lifter** |
| No plant growth regulator (NAA or IBA) | 3/18 (16.7%) |
| IBA (0.5 mg/L) | 2/9 (22.2%) |
| IBA (0.5 mg/L) with Activated Charcoal (0.5g/L) | 2/9 (22.2%) |
| NAA (0.5 mg/L)^b^ | 6/9 (66.7%) |
| NAA (0.5 mg/L) with Activated Charcoal (0.5g/L)^b^ | 9/9 (100.0%) |
| ^a^ Roots emerged within 8-10 days in all treatments  ^b^ Callus formation was observed prior to development or roots. | |

## Figure S1: Representative picture of heirloom varieties, some of which were used for implementing tissue culture and Agrobacterium transformation. A, M82. B, Sunray. C, Brandywine pink. D, Amana Orange. E, Jubilee. F, Mortgage lifter. Scale bars, 5 cm.

##
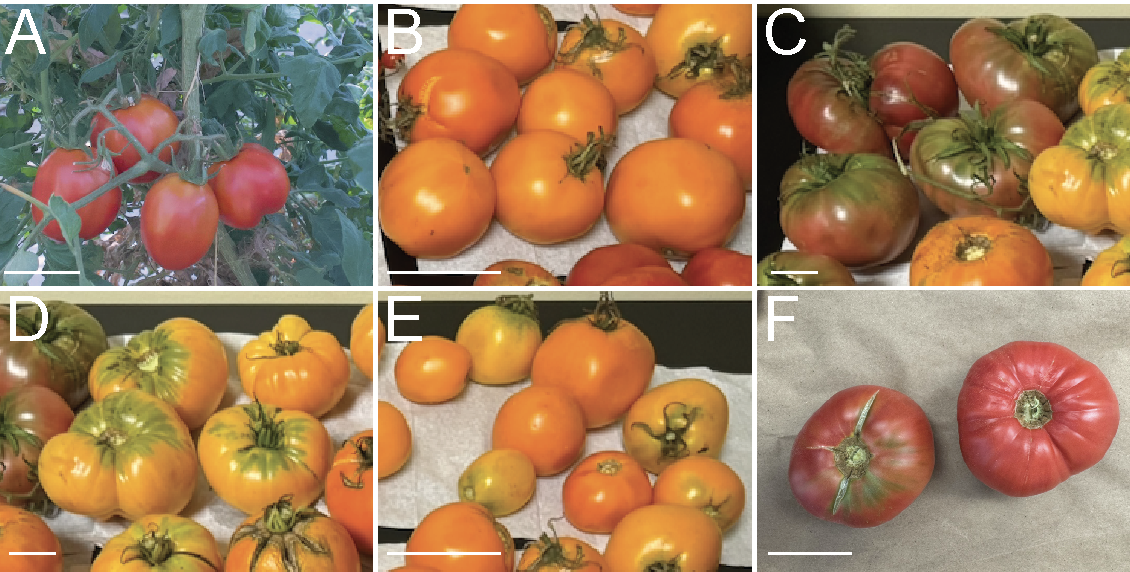


## Figure S2: Vector Architecture for plasmids pTL0047, pTL0151, and pTL0153. Vector architecture showing the expression cassettes contained within the left (LB) and right (RB) borders. pTL0047 contains the genes NPTII and Cas9. pTL0151 contains four guide RNAs (gRNAs) targeting the coding sequences of the genes *SP5G* and *Br*. pTL0153 was built by adding the gene *GRF4-GF1* and also contains the same gRNAs as pTL0151.


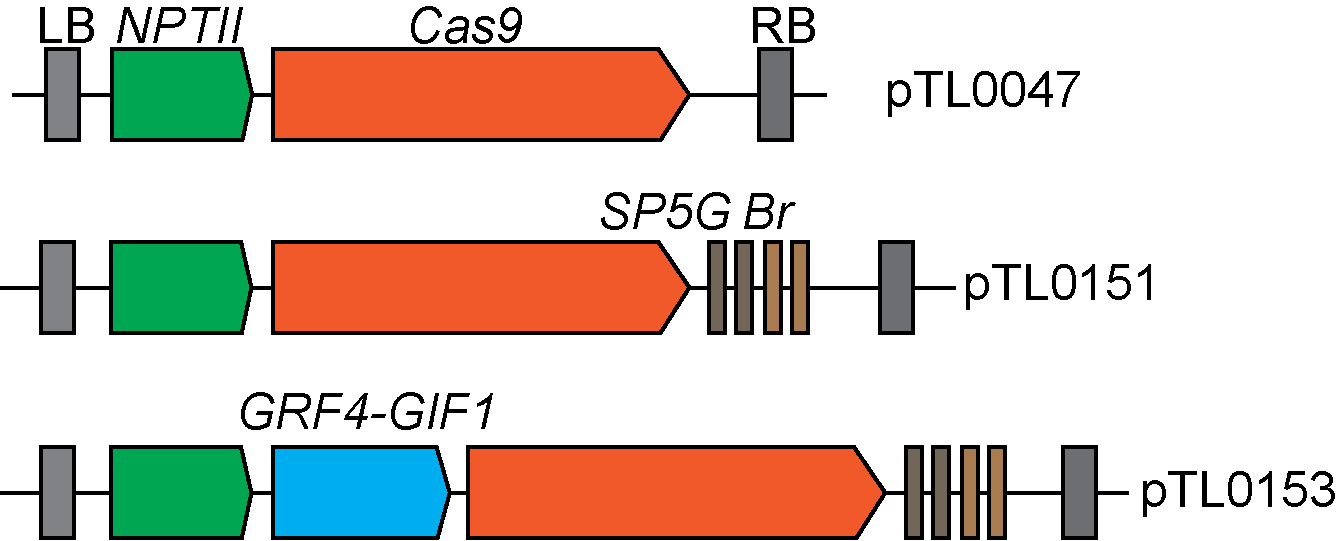


## Figure S3: Original gel electrophoresis showing the results from the genotyping by PCR performed on target genes *SP5G* and *Br*. The first gel picture represents the amplification of the *SP5G* target sites in selected T0 plants. The second gel represents the same samples, this time showing the amplification of the *BR* target sites.

##
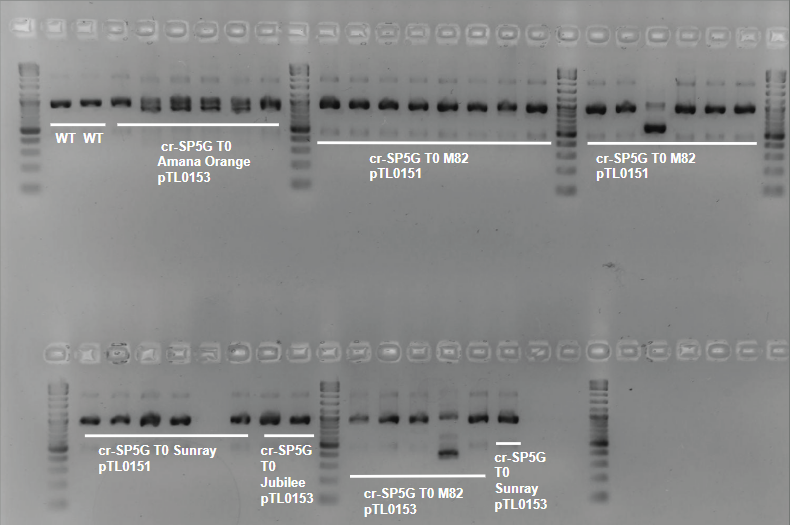


##
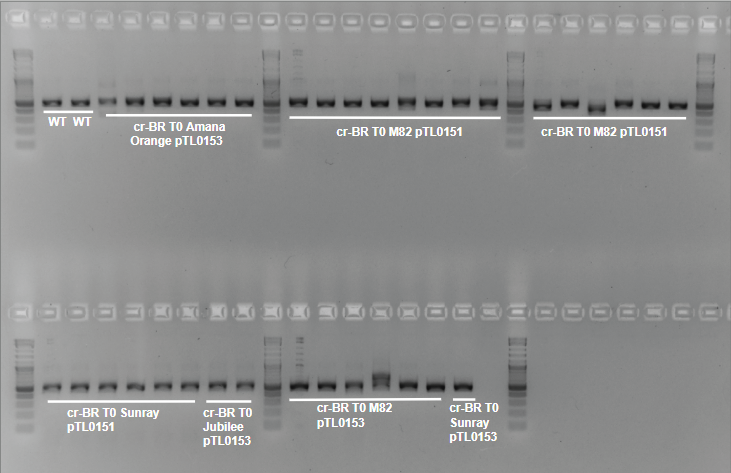

Supplement: Supplementary file 1 — Supplementary file1 (DOCX 5676 KB) [file 425_2026_5024_MOESM1_ESM.docx]
